# A Rare Morphological Variant of Postaxial Foot Polydactyly With an Accessory Articulating Osseous Structure of the Fifth Ray

**DOI:** 10.1155/cro/5110521

**Published:** 2026-08-27

**Authors:** Ali M. Aldossari, Ahmed K. Alanazi, Norah I. Alromaih, Sara N. Albqami

**Affiliations:** ^1^ Pediatric Orthopedic Surgery Consultant, King Saud Medical City, Riyadh, Saudi Arabia, ksmc.med.sa; ^2^ Orthopedic Surgery Resident, King Saud Medical City, Riyadh, Saudi Arabia, ksmc.med.sa

**Keywords:** foot anomaly, metatarsal duplication, postaxial polydactyly

## Abstract

Postaxial polydactyly of the foot is a common form of digital duplication that often causes difficulty with footwear, metatarsalgia, and cosmetic concerns. Although it may present as an isolated anomaly, it can also be associated with genetic conditions. Surgical excision is the primary intervention, with generally excellent outcomes. A 5‐year‐old male presented with postaxial polydactyly of the left foot, characterized by an accessory articulating osseous structure associated with the fifth ray. The main complaints were difficulty with footwear and cosmetic appearance. Radiographic evaluation revealed a shortened, dysplastic fifth metatarsal associated with an accessory osseous structure that articulates with the duplicated lateral digits. The anatomical configuration did not completely correspond to existing classification systems. Surgical excision of the supernumerary digit and realignment of the metatarsal head were performed. At the 24‐month follow‐up, the patient demonstrated satisfactory radiographic healing, maintained toe alignment, painless full weight‐bearing capacity, and an acceptable cosmetic appearance without postoperative complications. This case emphasizes the importance of detailed radiographic assessment and individualized surgical planning for rare anatomical variants of postaxial foot polydactyly that are not fully represented by current classification systems. Continued long‐term follow‐up is necessary to evaluate outcomes through skeletal growth.

## 1. Introduction

Polydactyly is the most frequent congenital anomaly affecting the forefoot, defined by the presence of supernumerary toes with or without metatarsal duplication. It is broadly categorized into preaxial, central, and postaxial types. Postaxial polydactyly—characterized by duplication adjacent to the fifth toe—is the most prevalent, accounting for 77%–85% of cases [1–3].

It is usually an isolated anomaly, but it may be associated with syndromes such as trisomy 13, tibial hemimelia, or Down syndrome [4]. Mainly, the management is surgical and aims to optimize function and appearance. In this report, we present a rare morphological variant of postaxial polydactyly characterized by an accessory articulating osseous structure of the fifth ray that does not completely correspond to existing classification systems.

## 2. Case Presentation

**Figure 1:**
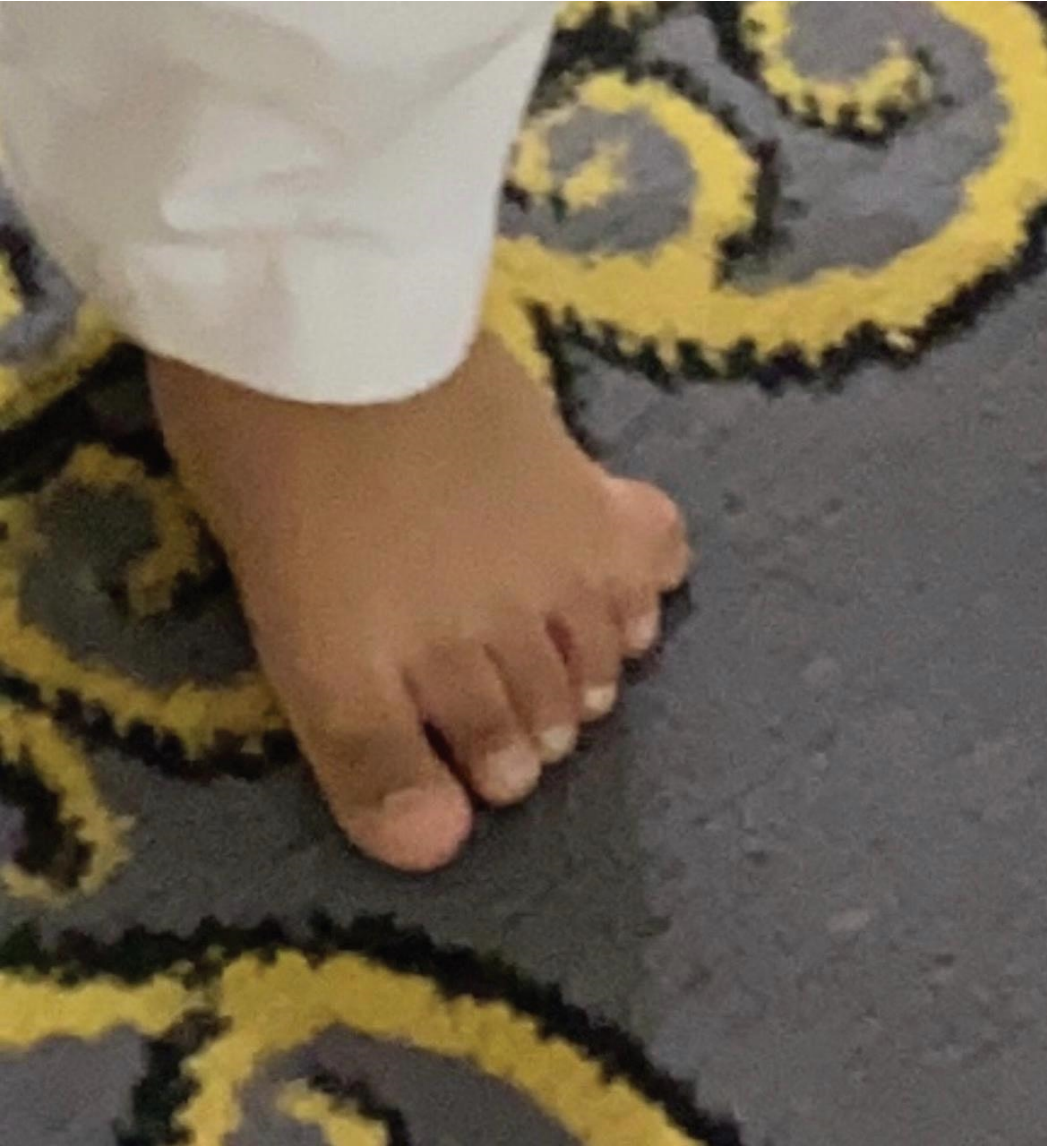
Clinical picture of left foot (preoperative).

A 5‐year‐old male was referred to our clinic by his parents for an isolated supernumerary fifth toe on the left foot (Figure 1). The patient had no concurrent medical comorbidities or congenital malformations. In addition, there was no known family history of polydactyly or other congenital limb anomalies. He had an unremarkable prenatal and postnatal history. The main issue was difficulty with shoe wear due to cutaneous irritation provoked by the additional digit. Parental concern also focused on the abnormal physical appearance.

**Figure 2:**
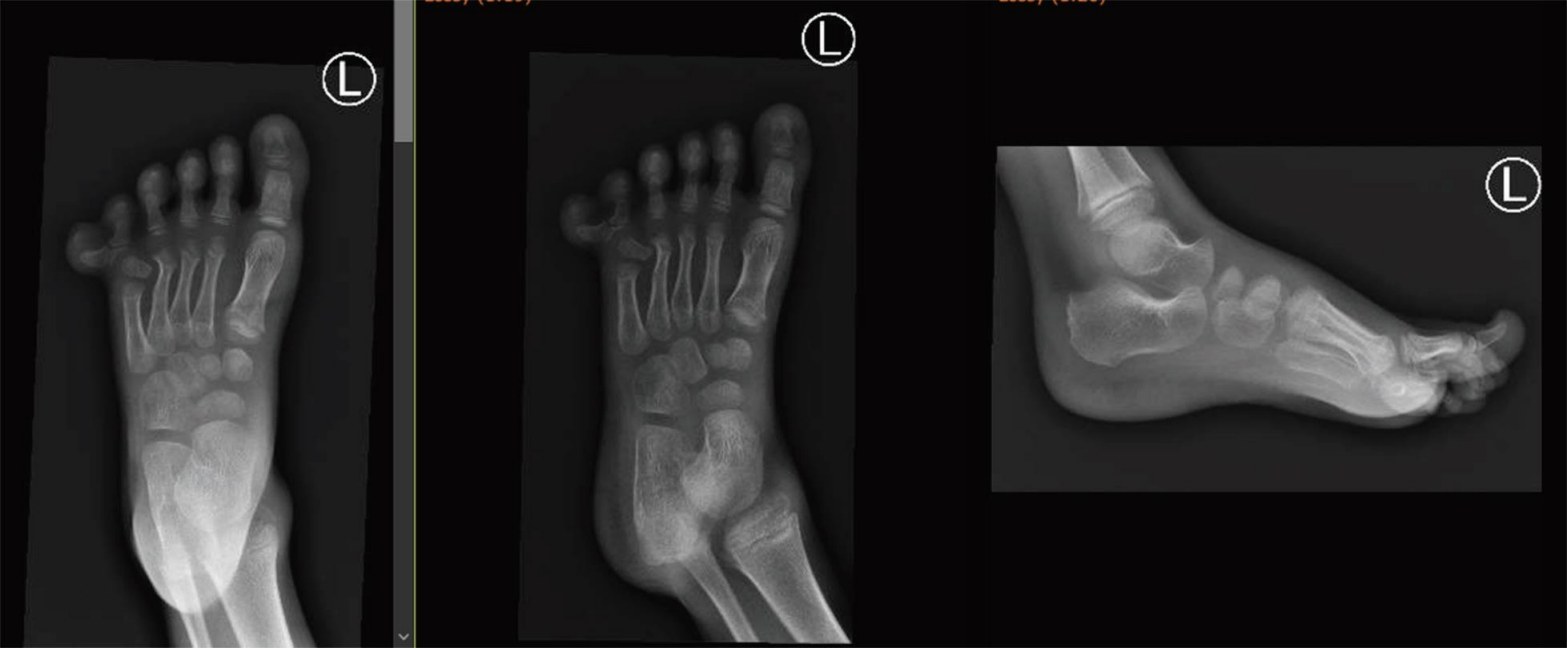
AP, oblique, and lateral view of the foot (preoperative).

We obtained radiographs of the child′s left foot and noted the following: (1) there was a complex postaxial duplication pattern involving the lateral ray; (2) the fifth metatarsal appeared shortened and dysplastic, with a formed distal epiphysis and widening of the distal metaphyseal region; (3) an accessory osseous structure was identified distal to the fifth metatarsal and articulated with both the fifth and supernumerary sixth toes; and (4) the fifth metatarsal lacked articulation with the proximal phalanx of the fifth toe. The oblique radiographic view best demonstrated the relationship between the accessory osseous structure and the duplicated digits. Given the unusual morphology and the lack of clear correspondence to established classification systems, the lesion was interpreted as an atypical accessory osseous duplication of the lateral ray (Figure 2). Based on the clinical and radiographic findings, the differential diagnosis included atypical postaxial polydactyly with metatarsal dysplasia and complex lateral‐ray duplication. Existing classification systems proposed by Watanabe, Lee, Seok, and Otsuka were reviewed; however, the accessory articulating osseous structure and abnormal fifth‐ray anatomy did not completely correspond to any recognized subtype. Therefore, individualized surgical planning was undertaken. The surgical intervention was discussed with the parents, who consented to proceed. The patient was then prepared for surgery. A chronological summary of the patient′s presentation, diagnostic evaluation, treatment, and follow‐up is provided in Table 1.

**Table 1:** The chronological clinical course of the patient.

Time	Clinical event
Birth	Left postaxial foot polydactyly noted
Age of 5 years	Presented to orthopedic clinic due to footwear difficulty and cosmetic concerns
Initial evaluation	Clinical examination and radiographs performed
Surgery	Surgical excision and reconstruction
2 weeks post op	Wound healed satisfactorily
6 weeks post op	Radiographic healing, weight‐bearing initiated
4 months post op	Maintained alignment and satisfactory healing
12 months post op	Good functional outcome
24 months post op	Good functional outcome

**Figure 3:**
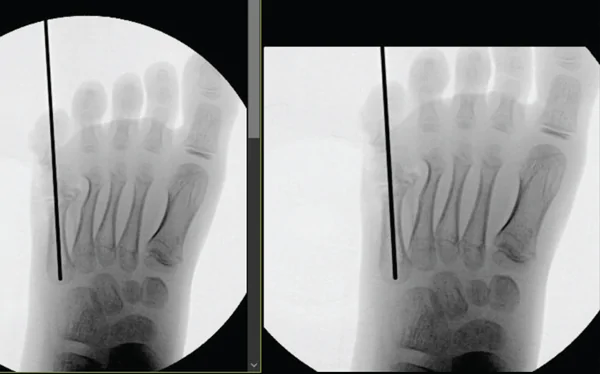
AP view of the foot (intraoperative).

**Figure 4:**
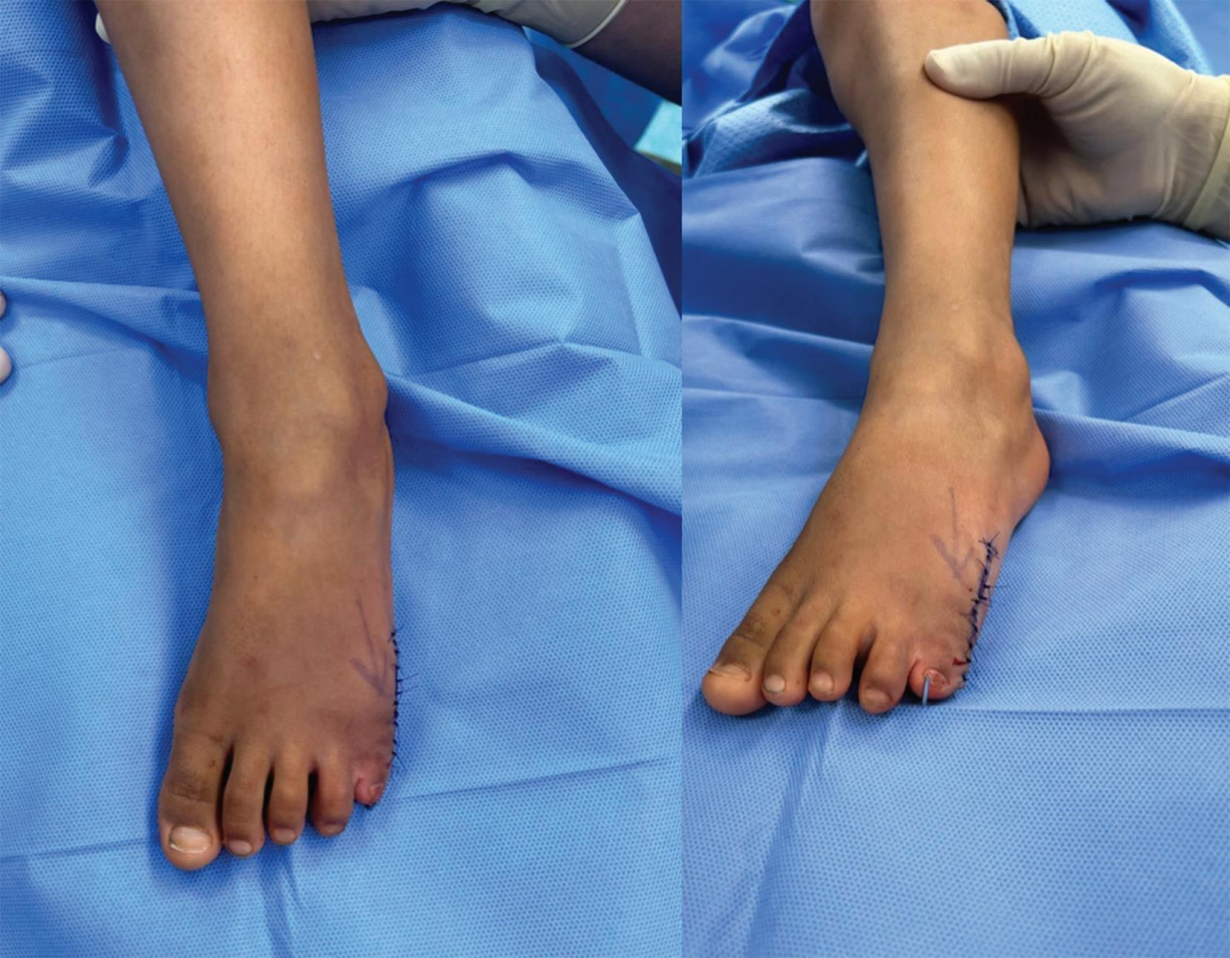
Clinical picture of left foot (intraoperative).

In the operating room, the patient was placed supine on a radiolucent table under general anesthesia. A thigh tourniquet was applied and inflated after preparation and draping. We made a circumferential racquet‐shaped incision over the extra toe. After incising the skin and subcutaneous tissue, we exposed the proximal phalanx and noted a shared physis (Salter–Harris type 4). We resected the extra toe and accessory osseous segment with a knife, then corrected the alignment of the metatarsal head with the distal phalanx using a K‐wire. The extra toe was used as an autograft at the distal fifth metatarsal site. We confirmed correction and excision under C‐arm imaging (Figure 3). The wound was irrigated with normal saline, the tourniquet was deflated, and closure was performed in layers—joint capsule, subcutaneous tissue, and skin—using absorbable sutures (Figure 4). Capillary refill was checked, and a below‐knee back slab was applied to reduce mobility and pain.

**Figure 5:**
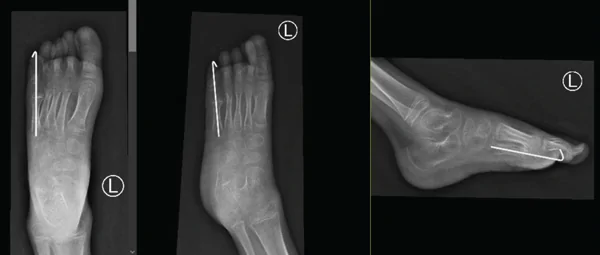
AP, oblique, and lateral view of foot (6 weeks follow‐up).

**Figure 6:**
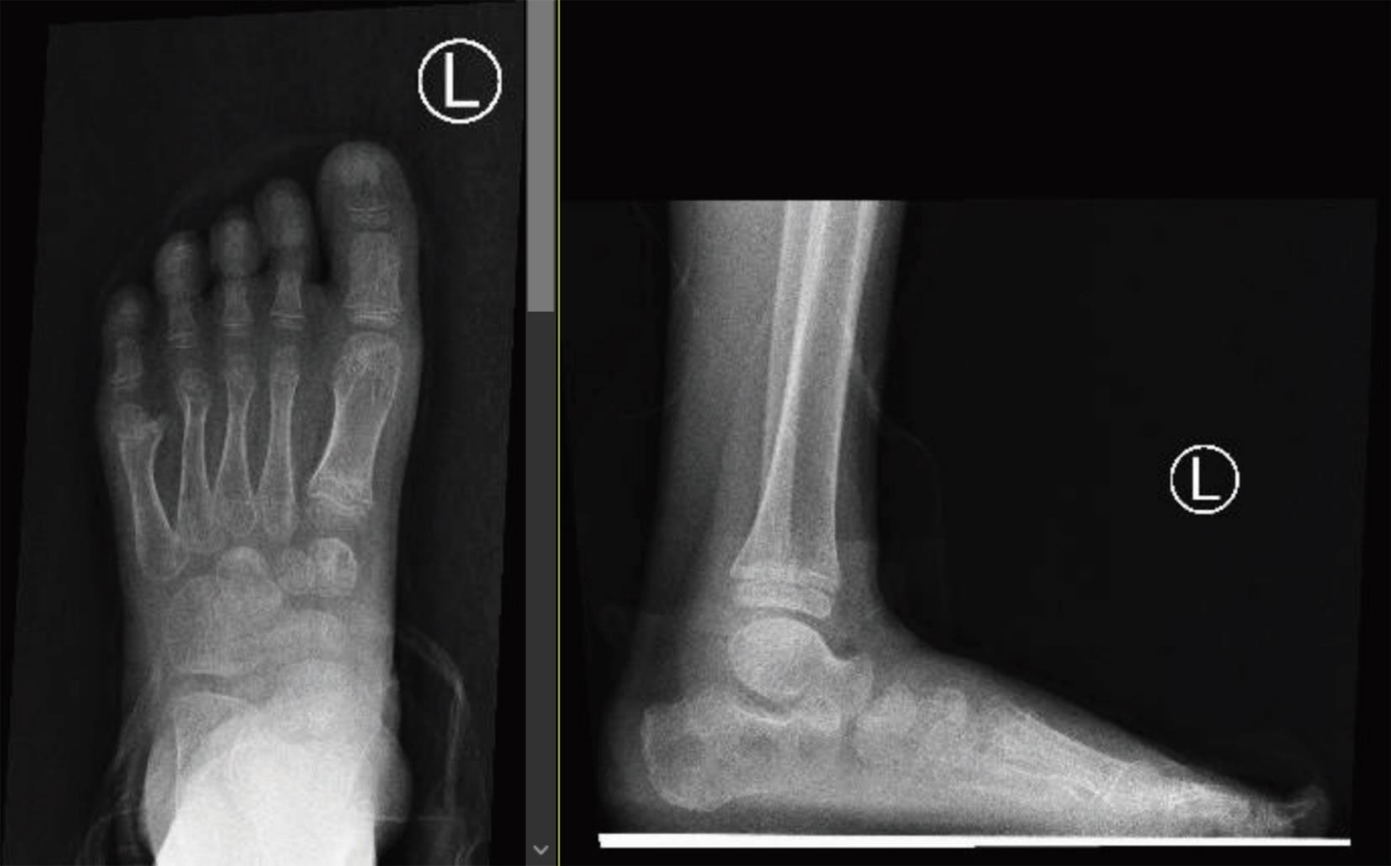
AP and lateral view of the foot (4 months follow‐up).

**Figure 7:**
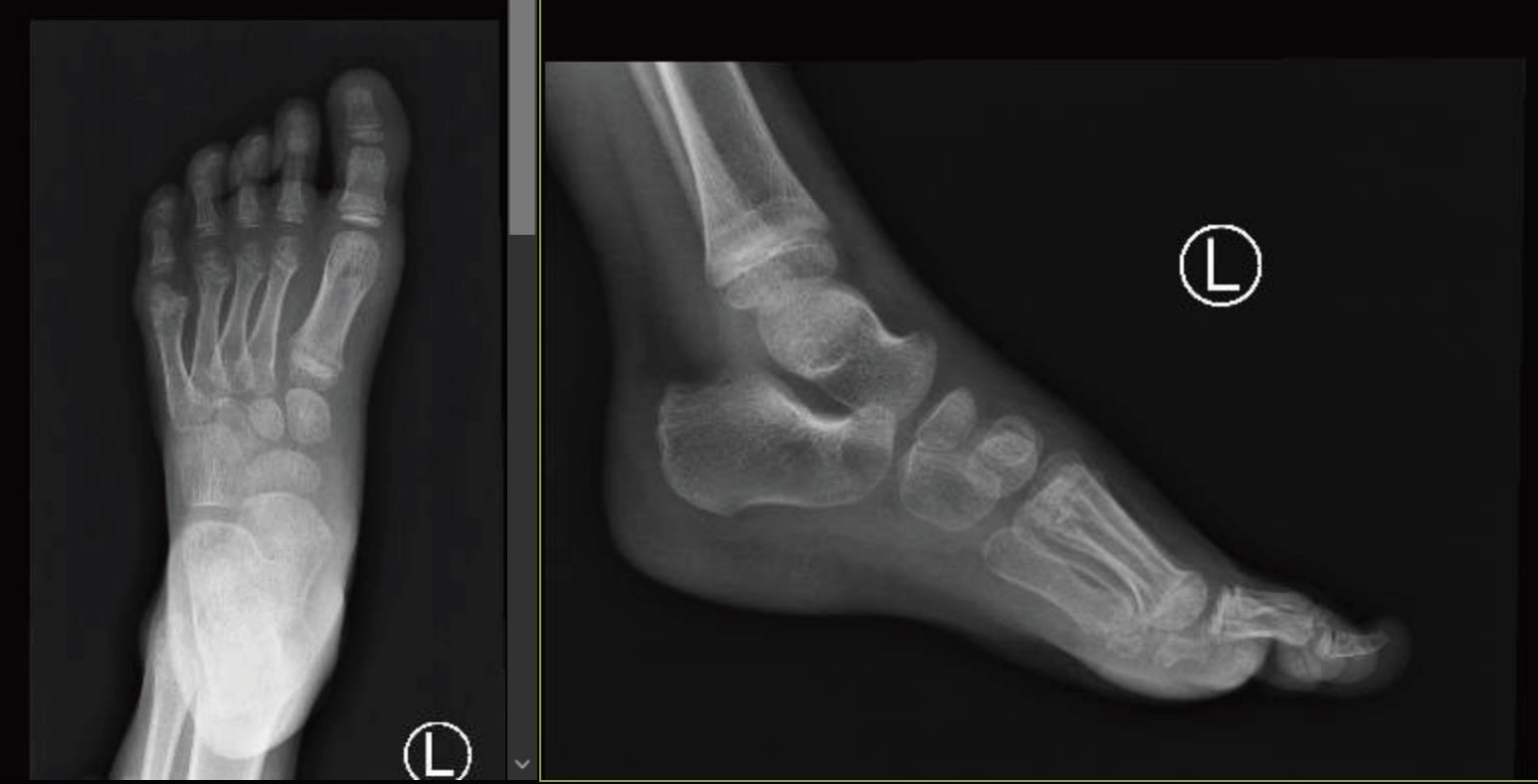
AP and lateral view of foot (12 months follow‐up).

**Figure 8:**
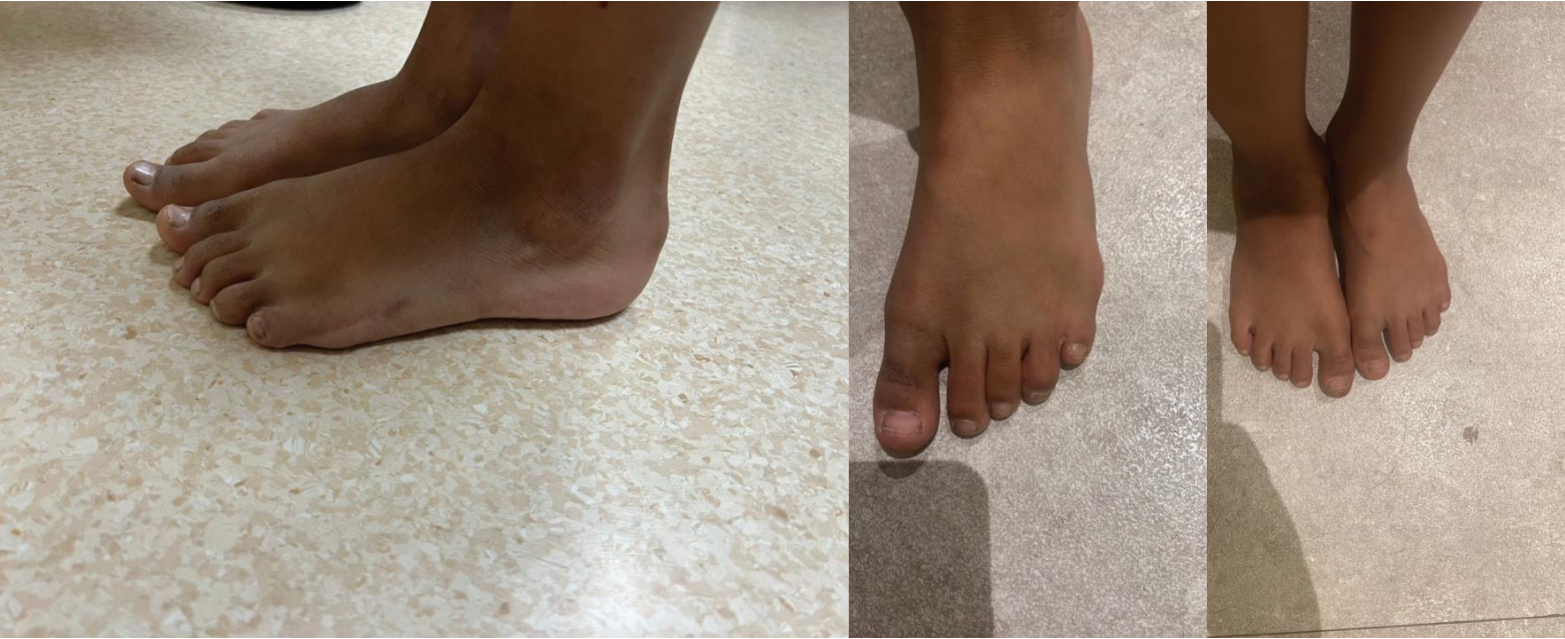
Clinical picture of left foot (12 months follow‐up).

**Figure 9:**
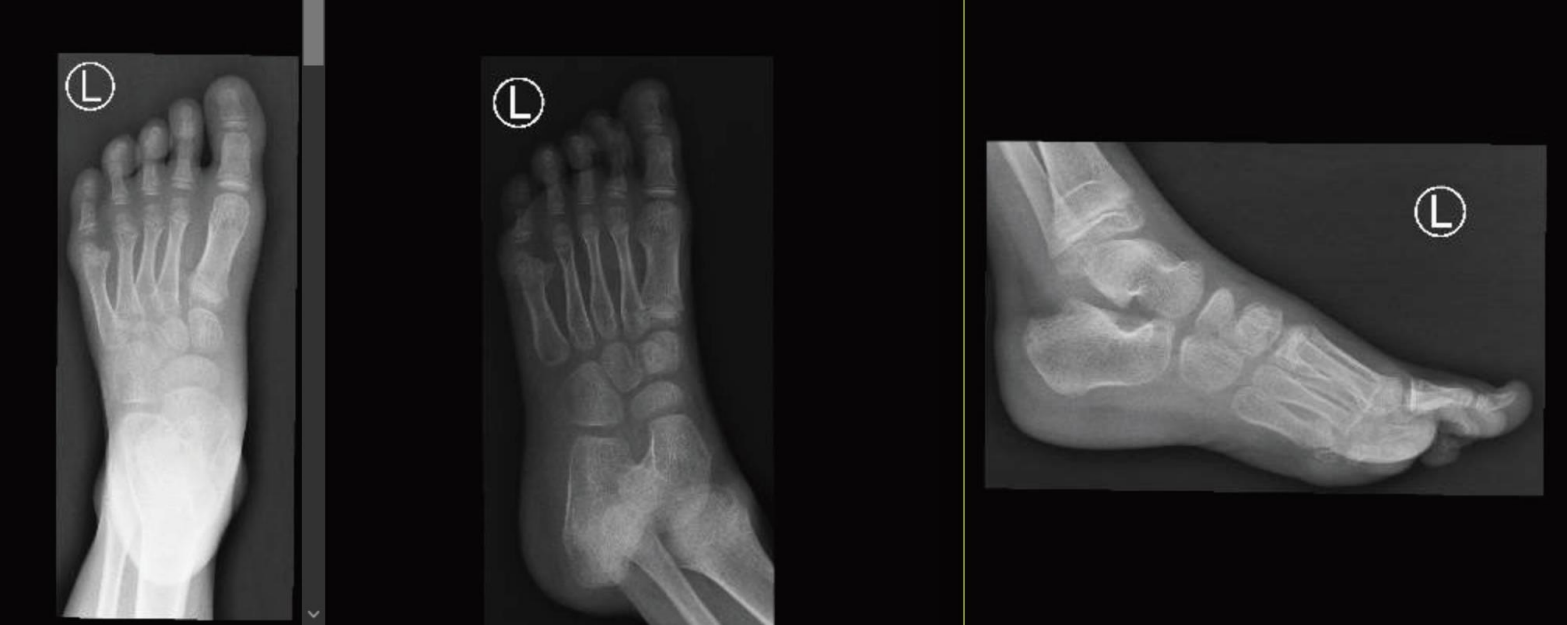
AP, oblique, and lateral view of foot (24 months follow‐up).

**Figure 10:**
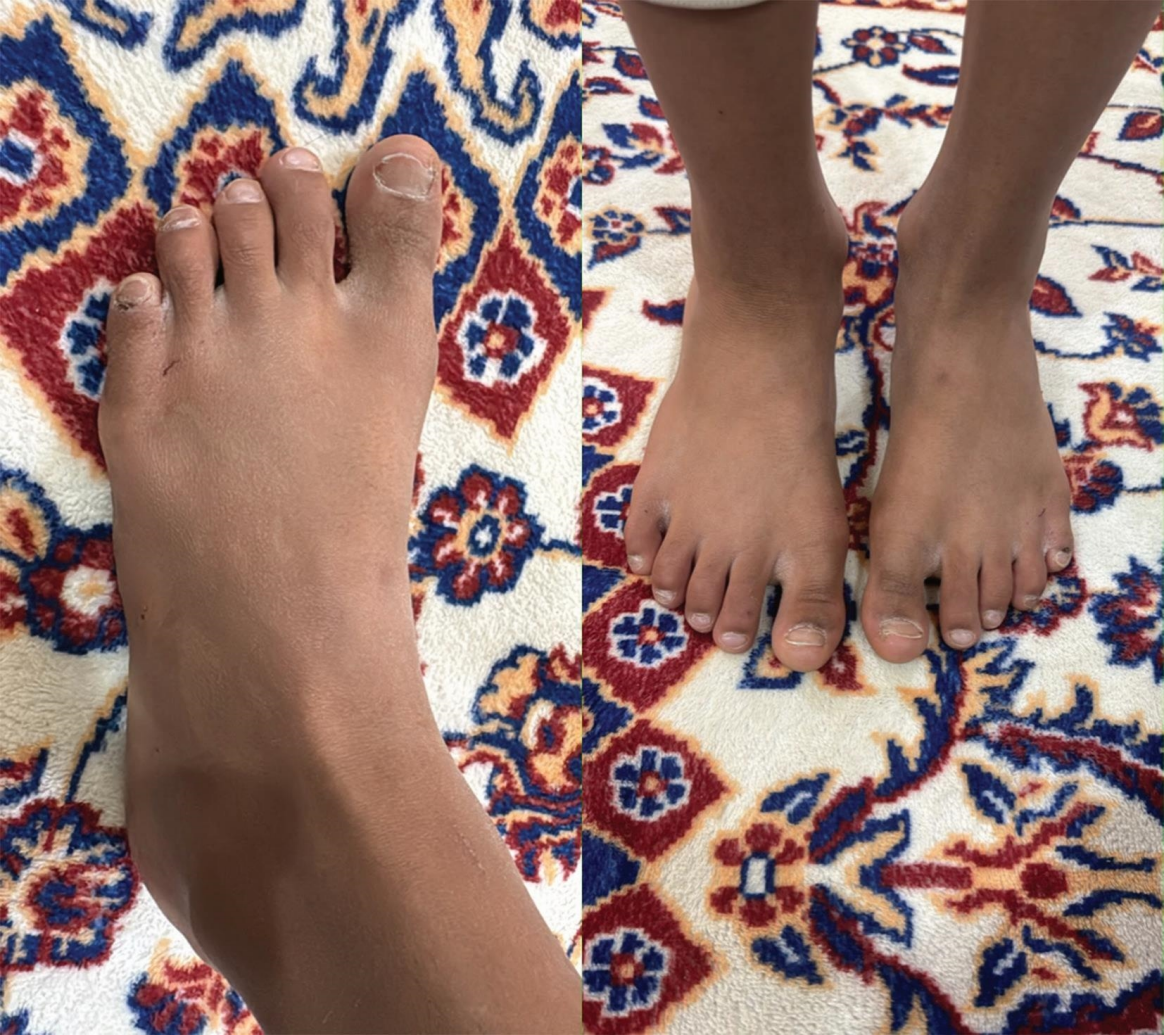
Clinical picture of left foot (24 months follow‐up).

Upon discharge, the patient was clinically stable with no immediate postoperative complications. Pain was adequately controlled, the surgical wound appeared clean with intact capillary refill to the toes, and there were no signs of vascular compromise or excessive swelling. The patient was maintained in a below‐knee back slab for immobilization and was instructed to remain nonweight‐bearing on the affected extremity until follow‐up evaluation. After 2 weeks, the patient and his parents presented to the clinic for a wound assessment, which revealed complete healing and intact capillary refill. At the sixth week, radiographs demonstrated satisfactory osseous healing (Figure 5), and weight‐bearing as tolerated was permitted. At 4 and 12 months postoperatively, both clinical and radiographic evaluations demonstrated acceptable cosmetic appearance, complete osseous healing, preserved toe alignment and function, and no evidence of postoperative complications (Figures 6, 7, and 8). At the 24‐month follow‐up, the patient demonstrated satisfactory radiographic healing, maintained toe alignment, painless full weight‐bearing, and an acceptable cosmetic appearance with good functional outcome, and the parents were satisfied with the outcomes (Figures 9 and 10).

## 3. Discussion

Postaxial polydactyly is one of the most frequently encountered congenital anomalies of the forefoot and is characterized by duplication along the lateral side of the foot [5, 6]. In many patients, it presents as an isolated finding; however, it may also occur as part of syndromic conditions such as Ellis–van Creveld syndrome, trisomy 13, trisomy 21, tibial hemimelia, and Greig cephalopolysyndactyly syndrome [4–6]. More complex forms of duplication, particularly those involving seven toes or polysyndactyly, are reported to be more strongly associated with syndromic conditions [7]. In isolated cases, a positive family history may be identified in up to 30% of patients [6]. Our patient had no associated congenital anomalies and no relevant family history.

Several classification systems have been introduced to better describe the wide spectrum of foot polydactyly. Watanabe et al. [8] classified cases according to anatomic location and the extent of metatarsal involvement, whereas Lee et al. [3] proposed five subtypes of postaxial polydactyly based on the duplicated skeletal structures. More recently, Seok et al. [9] developed a classification system that also accounts for syndactylism, axial deviation, and metatarsal involvement, with the goal of improving surgical planning and predicting postoperative alignment. Otsuka et al. [10] later proposed another morphology‐based classification focused on surgical outcomes in lateral foot polydactyly. Despite these efforts, some rare variants still do not fit neatly within existing systems.

In our patient, the fifth metatarsal appeared shortened and dysplastic, and was associated with an accessory osseous structure that articulated with the duplicated lateral digit. The morphology did not completely match any currently described subtype and therefore represented an atypical pattern of lateral ray duplication. Similar unusual cases have been described in the literature, including incomplete metatarsal duplications, complex polysyndactyly, and deformities requiring metatarsal transfer for reconstruction [7, 10, 11]. However, the accessory osseous articulation identified in our case resulted in a distinct anatomical configuration, making classification and surgical planning more challenging.

Patients with postaxial polydactyly commonly present because of cosmetic concerns, difficulty wearing shoes, skin irritation, or, less commonly, pain and gait disturbance [1, 12]. Radiographic evaluation plays a key role in identifying the exact skeletal anatomy, assessing joint alignment, and determining the dominant ray before surgery [6, 12]. Associated findings such as syndactyly, axis deviation, metatarsophalangeal malalignment, or abnormal metatarsal morphology can further increase the complexity of reconstruction [9, 10, 12].

The main goal of surgical treatment is to create a functional, stable, and cosmetically acceptable foot while preserving alignment and joint stability [1, 6, 13, 14]. In more typical forms of postaxial polydactyly, treatment usually involves excision of the less‐developed digit, along with soft‐tissue balancing and collateral ligament reconstruction [6, 13]. In our case, management required a more individualized approach due to the unusual accessory osseous articulation and abnormal fifth‐ray anatomy. Intraoperatively, careful assessment was needed to determine the optimal reconstruction strategy. Treatment included excision of the supernumerary digit and accessory osseous segment, realignment of the metatarsal head, and temporary stabilization with Kirschner‐wire fixation to maintain alignment and lateral column stability.

Overall, long‐term outcomes after surgical correction of foot polydactyly are generally favorable, with high rates of patient and parent satisfaction [1, 6]. Turra et al. [1] reported satisfaction rates exceeding 90%, with less favorable outcomes typically related to residual deformity, inadequate soft‐tissue reconstruction, hypertrophic scarring, or metatarsophalangeal instability. In our patient, recovery was uncomplicated, and follow‐up radiographs demonstrated satisfactory healing and alignment. However, residual prominence along the medial aspect of the reconstructed fifth metatarsal neck remains an important consideration. With continued skeletal growth, there is a theoretical risk of widening between the fourth and fifth toes or progressive divergence at the metatarsophalangeal joint. For this reason, long‐term clinical and radiographic follow‐up remains important to monitor for late deformity or functional symptoms.

Although classification systems and surgical techniques continue to evolve, uncommon variants such as the present case still pose challenges in both diagnosis and management [6, 9, 10]. Further studies focusing on long‐term functional outcomes, patient‐reported satisfaction, and advanced preoperative planning techniques may help improve the management of these rare deformities [6, 12].

In conclusion, postaxial polydactyly requires careful assessment of the underlying skeletal anatomy and an individualized surgical approach. Rare variants with atypical accessory osseous articulations may not fit within existing classification systems and, therefore, require flexible intraoperative decision‐making. In our case, detailed radiographic evaluation and case‐specific reconstruction resulted in a stable, functional, and cosmetically satisfactory outcome without complications during follow‐up. Continued follow‐up through skeletal maturity remains important.

## Author Contributions

The first, second, and third contributor prepared the first draft of the manuscript and treated the patient. All contributors conducted a literature search, contributed to preparation of the manuscript, and prepared the final manuscript.

## Funding

No funding was received for this manuscript.

## Ethics Statement

According to the policy of the Institutional Review Board (IRB) of King Saud Medical City, single‐patient case reports do not require formal IRB approval. The case was reviewed and determined to be exempt from ethical approval.

## Consent

Written informed consent was obtained from the patient′s parent for publication of this case report, including the clinical photographs and radiographic images.

## Conflicts of Interest

The authors declare no conflicts of interest.

## Data Availability

The data supporting the findings of this study are available within the article. Additional anonymized data are available from the corresponding author upon reasonable request.
